# Transcriptome Analysis of Cadmium-Treated Roots in Maize (*Zea mays* L.)

**DOI:** 10.3389/fpls.2016.01298

**Published:** 2016-08-31

**Authors:** Runqing Yue, Caixia Lu, Jianshuang Qi, Xiaohua Han, Shufeng Yan, Shulei Guo, Lu Liu, Xiaolei Fu, Nana Chen, Haiyan Yin, Haifeng Chi, Shuanggui Tie

**Affiliations:** ^1^Food Crops Research Institute, Henan Academy of Agricultural SciencesZhengzhou, China; ^2^The Henan Provincial Key Laboratory of Maize BiologyZhengzhou, China

**Keywords:** auxin, auxin transport, cadmium, differentially expressed genes, maize, transcriptome

## Abstract

Cadmium (Cd) is a heavy metal and is highly toxic to all plant species. However, the underlying molecular mechanism controlling the effects of auxin on the Cd stress response in maize is largely unknown. In this study, the transcriptome produced by maize ‘Zheng 58’ root responses to Cd stress was sequenced using Illumina sequencing technology. In our study, six RNA-seq libraries yielded a total of 244 million clean short reads and 30.37 Gb of sequence data. A total of 6342 differentially expressed genes (DEGs) were grouped into 908 Gene Ontology (GO) categories and 198 Kyoto Encyclopedia of Genes and Genomes terms. GO term enrichment analysis indicated that various auxin signaling pathway-related GO terms were significantly enriched in DEGs. Comparison of the transcript abundances for auxin biosynthesis, transport, and downstream response genes revealed a universal expression response under Cd treatment. Furthermore, our data showed that free indole-3-acetic acid (IAA) levels were significantly reduced; but IAA oxidase activity was up-regulated after Cd treatment in maize roots. The analysis of Cd activity in maize roots under different Cd and auxin conditions confirmed that auxin affected Cd accumulation in maize seedlings. These results will improve our understanding of the complex molecular mechanisms underlying the response to Cd stress in maize roots.

## Introduction

Cadmium (Cd) is a highly toxic, non-essential element. It inhibits plant growth and development through its effect on physiological and metabolic processes, including growth reduction, leaf roll and chlorosis, respiration, photosynthesis, uptake competition, plant antioxidant defenses, generation of oxidative stress and lipid peroxidation, damage to the cell membrane, and enzyme inhibition (Hasan et al., 2009; DalCorso et al., 2010; Andresen and Kupper, 2013). Cadmium contamination is a major ecological concern due to its widespread release by industry and other human activities (Valko et al., 2005). Over the last few decades, Cd-contaminated soil has dramatically increased worldwide, and Cd accumulation in crops poses a potentially significant threat to human health (Grant et al., 2008; Uraguchi et al., 2009).

Recently, an increasing number of studies have revealed the involvement of various phytohormones in plant responses to Cd stress (Sneideris et al., 2015). In *Arabidopsis*, an application of brassinosteroids to wide type plants significantly enhanced Cd-induced root growth inhibition, which showed that there was a functional interaction between brassinosteroid signaling and Cd (Villiers et al., 2012). In tomato, the role of brassinosteroids in the alleviation of Cd-induced oxidative stress and photosynthetic inhibition has also been revealed (Hasan et al., 2011; Ahammed et al., 2013). Cd-induced endogenous salicylic acid enhances tolerance to Cd stress by regulating the rate-limiting step in plant glutathione synthesis (Guan et al., 2015). Gibberellic acid (GA), an important phytohormone involved in plant responses to abiotic stresses, alleviates Cd toxicity by reducing Cd-dependent NO accumulation and Cd^2+^ uptake related gene expression in *Arabidopsis* (Zhu et al., 2012), whereas exogenous methyl jasmonate inhibits the uptake of Cd to the aboveground part of *Kandelia obovata* seedlings (Chen et al., 2014). Ethylene is a regulator of multiple plant processes and Cd induces the biosynthesis of ethylene in *Arabidopsis* mainly via the increased expression of ACS2 and ACS6 (Schellingen et al., 2014).

In addition, a close relationship between auxin and Cd stress has been reported in several plant species. In barley, Cd-induced mild oxidative stress causes root growth inhibition by regulating the IAA signaling in root tip (Tamas et al., 2012). In rice, auxin signal modification plays a major role in the expression of cell-cycle genes under Cd stress (Zhao et al., 2012). In poplar, Cd stress interferes with auxin physiology and lignifications by triggering increases in the activities of GH3 enzymes (Elobeid et al., 2012). In maize, the expression patterns of *GH3* genes are responsive to several abiotic stresses including Cd treatment. Cd stress suppresses free IAA contents suggesting a interaction between Cd and GH3-mediated auxin levels in maize roots (Feng et al., 2015).

Maize is widely cultivated cereal and tolerant to the Cd-contaminated soils (Van Slycken et al., 2013). Maize has been used as an optimum plant for Cd phytoremediation in contaminated soils (Meers et al., 2010; Xu et al., 2014). Recently, an increasing number of transcriptome studies screened out a series of candidate genes involved in the responses to Cd stress in various plant species (Milner et al., 2014; Oono et al., 2014; Chen S.F. et al., 2015; Gao et al., 2015; Oono et al., 2016). A transcriptome data of maize roots response to Cd pollution has been already published by Peng et al. (2015). *Zea mays* L. inbred line Zheng 58, a RBSDV (rice black-streaked dwarf virus)-resistant inbred line widely planted in central China, was used in our study. Based on the transcriptome data, auxin pathway-related genes were found to be regulated, so we have focused on analyzing them in more detail. Our study provides fundamental information on the candidate genes and auxin transportation involved in the responses to Cd stress in this major cultivated maize variety in central China.

## Materials and Methods

### Plant Growth Conditions and Cadmium Stress Treatment

The maize (*Zea mays* L. inbred line Zheng 58) seeds were surface sterilized, washed with ddH_2_O, and then germinated overnight in an incubator at 30°C. The seedlings were planted in a growth chamber with a photoperiod of 16-h light/8-h dark and a relative humidity of 60%. A half-strength, modified Hoagland nutrient solution (pH = 5.8) was used and changed every 3 days. The seedlings were grown in two groups of 40 × 40 cm pots (10 seedlings per pot) representing different treatments. Two-week-old seedlings were grown in nutrient solution with or without 100 μM cadmium chloride for 7 days. Then the root samples were harvested and stored at -80°C in preparation for further assays. Total RNA was extracted from the different samples using TRIzol reagent (Invitrogen, Carlsbad, CA, USA) following the manufacturer’s protocol. RNA contamination was detected using a Bioanalyzer 2100 and RNA 6000 Nano LabChip Kit (Agilent, Santa Clara, CA, USA) and removed by 1% agarose gel electrophoresis.

### Construction and Sequencing of the mRNA Library

Approximately 10 μg of total RNA was subjected to isolate poly (A) mRNA with poly-T oligo attached magnetic beads (Invitrogen, Beijing, China). Following purification, the mRNA was fragmented into small pieces using divalent cations at elevated temperatures. Then the cleaved RNA fragments were reverse-transcribed to create the final cDNA library in accordance with the protocol for the mRNA-Seq sample preparation kit (Illumina, San Diego, CA, USA). The average insert size for the paired-end libraries was 300 bp (±50 bp). Then we performed paired-end sequencing on an Illumina Hiseq2000/2500 (LC Sciences, USA) following the manufacturer’s protocol. Six RNA libraries consisted of three control libraries and three Cd-treated libraries.

### Sequence and Primary Analysis

We used the Illumina paired-end RNA-seq approach to sequence the maize root transcriptome, which generated 244 million paired-end reads. Three repetitions of each treatment have been sequenced. This yielded 30.37 Gb of sequences, which was approximately 13.2 times the size of the genome (2.3 Gb) (Schnable et al., 2009). Prior to assembly, low quality reads, including reads containing sequencing adaptors, sequencing primers, and nucleotides with a q quality score lower than 20, were removed. The raw sequence data have been submitted to the NCBI Short Read Archive with an accession number of GSE74516.

### RNA-seq Reads Mapping

We aligned the reads of the different samples to the MaizeGDB^1^ maize reference genome using Tophat package v2.0.9 (Trapnell et al., 2010), which initially removes a proportion of the reads based on the quality information accompanying each read, and then maps the reads to the reference genome. Tophat allows multiple alignments per read and a maximum of two mismatches when mapping the reads to the reference genome. Tophat builds a database of potential splice junctions and confirms these by comparing the previously unmapped reads against the database for putative junctions.

### Transcript Abundance Estimation and Differential Expression Testing

The aligned read files were processed by Cuﬄinks, which uses the normalized RNA-seq fragment counts to measure the relative abundances of the transcripts. Cuﬄink was used to de novo assemble the transcriptome, and then Cuffmerge was used to integrate all the transcripts from the different samples to generate unique transcripts. The final unigenes that showed differential expressions between the different maize treatments were detected by DEGseq software using three replicates per sample (Anders and Huber, 2010). The unigene expression levels were calculated using reads per kilobase per million reads (RPKM), which eliminated the influences of gene length and sequencing level during the calculation of gene expression. A general Chi-squared test of statistical significance was used, and the false discovery rate (FDR) for the results was controlled (FDR < 0.05) (Ran and Peng, 2016). Significantly altered genes were described using heatmap analysis with unsupervised hierarchical clustering. The raw intensity (RPKM) was log_2_ transformed and then used to calculate the *Z* scores (Cheadle et al., 2003).

### Gene Annotation, Classification, and Metabolic Pathway Analysis

To assign putative functions to differentially expressed genes (DEGs) during maize responses to the treatments, various bioinformatics approaches were used for further annotation, classification, and metabolic pathway analysis. First, the DEGs were aligned to the web-based agriGO tools. GO enrichment analysis of DEGs was implemented using singular enrichment analysis (SEA) by comparing a query list of DEGs to a background gene set (FDR < 0.05). The SEACOMPARE tool was used for a comparative analysis that integrated the SEA cross-comparison. Finally, KOBAS software was used to test DEG statistical enrichment in the KEGG pathways (Xie et al., 2011).

### Quantitative Real-Time PCR Validation

Several DEGs with putative functions were selected randomly and validated by qRT-PCR to confirm the results of the RNA-Seq. The primers used in the qRT-PCR experiments were designed by Primer5 software and are listed in Supplementary Table S1. The methods, including RNA extraction from the maize seedling root samples, reverse transcription, and qRT-PCR, were performed according to the manufacturer’s protocols (Clontech, Dalian, China). Briefly, 1 μl of a 1/10 dilution of cDNA in double distilled water was added to 5 μl of 2× UltraSYBR. Then 100 nM of each primer was added to water to make up a final volume 10 μl. The procedure for the PCR were as follows: 95°C for 10 min; 40 cycles of 95°C for 15 s, and 60°C for 60 s. Heat map representation was performed using the average *Ct* value, and ClustalW software and Treeview were used to visualize the qRT-PCR analysis data. Five biological repeats were used for the expression analyses and the values shown in the figures represent the average values of these five repeats.

### Cd Content Determination and *In situ* Localization

Five seedlings for each treatment were oven-dried at 80°C for 3 days and then digested with nitric-perchloric acid (3:1, v/v) at 100°C. A microwave oven (LWY-84B Shenglan, Jiangsu, China) equipped with an infrared ray generation device was used to eliminate any interfering organic substances. The Cd content measurements were made by a graphite furnace atomic absorption spectrophotometer (ICE^TM^ 3300 AAS, ThermoFisher, USA).

After 7 days of treatment, the dithizone method (Balestri et al., 2014) was used to histochemically detect the Cd in the maize roots. The reddish colored precipitates produced by the dithizone-Cd reaction were determined and analyzed, and the results were used to localize the heavy metal Cd in the maize roots under the different treatments. Five independent roots were collected from each treatment, and the samples were stained for 1.5 h with a dithizone working solution (30 mg dissolved in 60 ml acetone and 20 ml distilled water), washed in clean water, and immediately analyzed using a Carl Zeiss LSM510 laser scanning system.

### Hormone Treatments and IAA Contents Measurements

For hormone treatments, 2-week-old seedlings were transferred to hormone free nutrient solution or nutrient solution with 0.1 μM 1-Naphthaleneacetic acid (NAA) and 1 μM 1-Naphthoxyacetic acid (1-NOA) for 7 days respectively. For Cd treatment, 2-week-old seedlings were grown in nutrient solution with 50 μM cadmium chloride for 7 days. For hormone-Cd combined treatments, 2-week-old seedlings were grown in 0.1 μM NAA + 50 μM cadmium chloride for 7 days or 1 μM 1-NOA + 50 μM cadmium chloride for 7 days. Seedlings in hormone free and Cd free nutrient solution were used as control.

The root samples from control and Cd-treated seedlings were cut and homogenized by 50 mM Tris-HCl buffer, pH 7.6. Then, samples were collected by centrifugation at 12,000 *g* in a 1.5 ml centrifuge and keep in liquid nitrogen immediately. Five independent biological replicates of 20 mg each were purified after addition of 250 pg of ^13^C6-IAA internal standard using ProElu C18^2^, Auxin content were measured with FOCUS GC-DSQII (Thermo Fisher Scientific Inc., Austin, TX, USA). The IAA oxidase activity was determined according to Xu et al. (2010).

### Statistical Analysis

Differences between values were calculated using one-way analysis of ANOVA with Student’s *t*-test at a significance level of 0.05 in Excel software. All experiments were performed for five biological repeats and the values shown in figures represent the average values of five repeats.

## Results

### Transcriptome Sequencing of the Control and the Treated ‘Zheng 58’ Seedling Roots

The global gene expression profiles were surveyed using the RNA-Seq approach in order to identify the DEGs that were responsive to Cd stress in the maize seedling roots. The raw Illumina sequencing reads were qualified and adapter trimmed to yield a total of 244 million clean short reads, which contained 30.37 Gb of sequence data from six complementary cDNA libraries. Over 98.76% of the clean reads had quality scores at the Q20 level and over 83% of the clean reads had scores at the Q30 level. A high proportion of the valid, clean reads (53.48–66.24%) were readily mapped onto the maize reference genome sequence in B73 after the different treatments (Supplementary Table S2). The transcriptional abundances of the genes were quantified using Cuﬄinks and measured as RPKM. A total of 88,557 transcripts have been identified in maize by previous studies, of which, 85,663 transcripts were expressed in our samples.

### Transcriptional Changes in Response to Cd Stress

**FIGURE 1:**
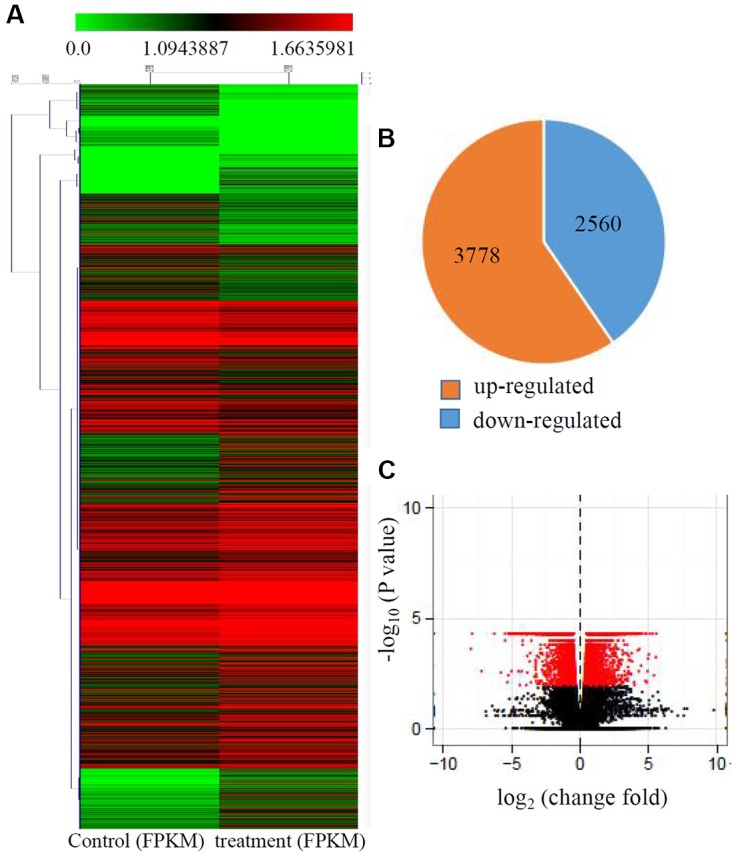
**Transcriptional changes in response to Cd stress. (A)** Expression profiles of the differentially expressed genes response to Cd stress were analyzed and clustered by K-means method. Red indicates up-regulated genes and green indicates down-regulated genes. **(B)** The numbers of up-regulated genes and down-regulated genes under Cd treatment. **(C)** Volcanoplots of the DEGs between control and the treated roots.

Transcriptional changes in response to Cd stress were determined by comparing the control and treated maize seedlings transcriptomes using cuffdiff software. The global comparisons of the gene expression profiles for the control and Cd treated samples are shown in **Figure 1A**, and differences in the expression levels between the two samples are shown as log_2_-transformed ratios. After using the 1.5-fold and padj < 0.01 criteria to select genes, 6342 genes, including 3778 induced genes and 2560 down-regulated genes, were identified as being differentially expressed after Cd treatment (**Figures 1B,C**).

**FIGURE 2:**
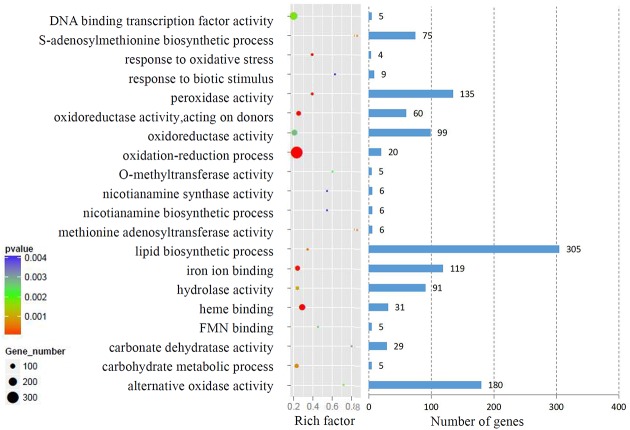
**Gene ontology (GO) term enrichment analysis of DEGs response to Cd treatment.** The top 20 enriched GO terms were showed.

**FIGURE 3:**
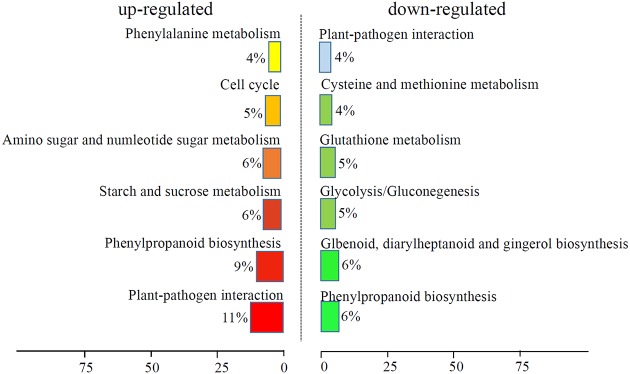
**KEGG analysis of DEGs response to Cd treatment.** The top six enriched KEGG terms of up-regulated and down-regulated DEGs were showed.

We analyzed the GO terms represented by these genes to obtain useful information about the DEG responses to Cd treatment. In total, 908 enriched GO terms were identified within the DEGs. GO term enrichment analysis indicated that various biological processes and molecular functions, such as DNA binding transcription factor, *S*-adenosylmethionine biosynthesis, response to oxidative stress, response to biotic stimulus, and peroxidase activity, were significantly enriched in the DEGs (**Figure 2**; Supplementary Table S3). We mapped the DEGs to the reference canonical pathways in the Kyoto Encyclopedia of Genes and Genomes (KEGG) to further identify the active metabolism pathways involved in the responses to Cd. All DEGs could be classified into 198 predicted biosynthesis pathways, of which, 22 metabolic pathways were significantly enriched (*p* < 0.05) (Supplementary Table S4). A high proportion of the up-regulated DEGs were enriched in six KEGG pathways. These were plant-pathogen interaction (11%), phenylpropanoid biosynthesis (9%), starch and sucrose metabolism (6%), amino sugar and nucleotide sugar metabolism (6%), cell cycle (5%), and phenylalanine metabolism (4%). In contrast, a large number of down-regulated DEGs were also enriched in the phenylpropanoid biosynthesis (6%), glbenoid, diarylheptanoid and gingerol biosynthesis (6%), glycolysis/gluconegenesis (5%), glutathione metabolism (4%), cysteine and methionine metabolism (4%), and plant-pathogen interaction (4%) KEGG pathways (**Figure 3**).

### Expression Changes in the Auxin Signaling Pathway Genes under Cd Treatment

**FIGURE 4:**
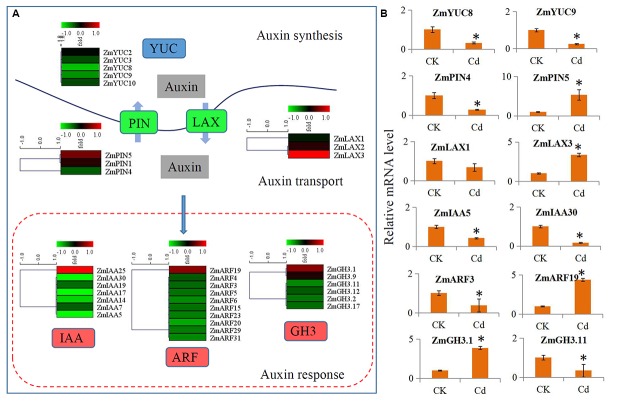
**Transcript abundance changes of auxin signaling-related genes in maize response to Cd stress. (A)** Expression changes of the genes associated with auxin synthesis, auxin transport and auxin response. Red indicates up-regulated genes and blue indicates down-regulated genes. **(B)** Real-time quantitative PCR validation of several selected auxin-related genes in maize response to Cd stress. Significant (*P* < 0.05) differences between CK and Cd treatment are indicated by an asterisk.

The expressions of the auxin signaling pathway genes were analyzed to determine the involvement of auxin and auxin signaling in the maize response to Cd. In our study, a large number of auxin-related genes were identified as DEGs. Comparison of the transcript abundances for auxin biosynthesis, transport, and downstream response genes revealed a universal expression response under Cd treatment (**Figure 4A**). Five biosynthesis-related genes (*ZmYUC2*, *ZmYUC3*, *ZmYUC8*, *ZmYUC9*, and *ZmYUC10*) were significantly down-regulated by Cd treatment; the *ZmPIN1* and *ZmPIN5* auxin eﬄux carriers were up-regulated; and *ZmPIN4* was down-regulated. The *ZmLAX2* and *ZmLAX3* auxin influx carriers were induced by Cd treatment; whereas *ZmLAX1* was reduced by Cd treatment. Interestingly, only *ZmIAA25*, *ZmARF19*, *ZmGH3.1*, and *ZmGH3.9* were up-regulated by Cd treatment, and most of the auxin downstream response genes were down-regulated by Cd treatment.

We performed qRT-PCR assays with independent samples (roots from the control and Cd treated seedlings) to verify the DEGs related to auxin signaling that were identified using RNA-Seq. We randomly selected 12 unigenes from the auxin signaling pathway to validate the RNA-Seq data. The expression levels of these selected genes were basically consistent with the RNA-Seq results (**Figure 4B**).

### Effects of Cd Stress on IAA Content and the Activity of IAA Oxidase in Maize Seedlings

**FIGURE 5:**
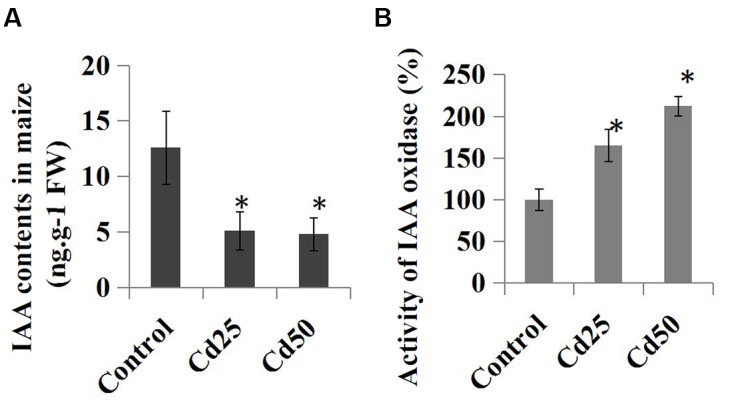
**Effects of Cd stress on the auxin content and activity of IAA oxidase in maize ‘Zheng 58’ roots. (A)** Two-week-old maize seedlings were treated with 25 μM Cd and 50 μM Cd for 7 days and then used for IAA content determination. **(B)** Two-week-old maize seedlings were treated with two different concentrations of Cd (25 and 50 μM) for 7 days and used for IAA oxidase activity determination. The data were analyzed by three independent repeats, and standard deviations were shown with error bars. Significant differences in IAA contents and IAA oxidase activity between control and the treated-roots were indicated by “^∗^.”

We further examined the effects of Cd stress on auxin levels and the endogenous IAA contents in the control and Cd-treated maize seedlings. Compared to the control roots, treatment with 25 μM Cd reduced the endogenous IAA content from 12.6 ng.g^-1^ to 5.1 ng.g^-1^, and the IAA content was reduced to 4.8 ng.g^-1^ by 50 μM Cd (**Figure 5A**). Furthermore, the relative IAA oxidase activities were measured in the Cd-treated samples and compared to the control samples. The results suggested that IAA oxidase activities were clearly induced by the 25 μM Cd and 50 μM Cd treatments (**Figure 5B**).

### Involvement of Auxin Signaling in the Root Growth of Maize under Cd Stress

**FIGURE 6:**
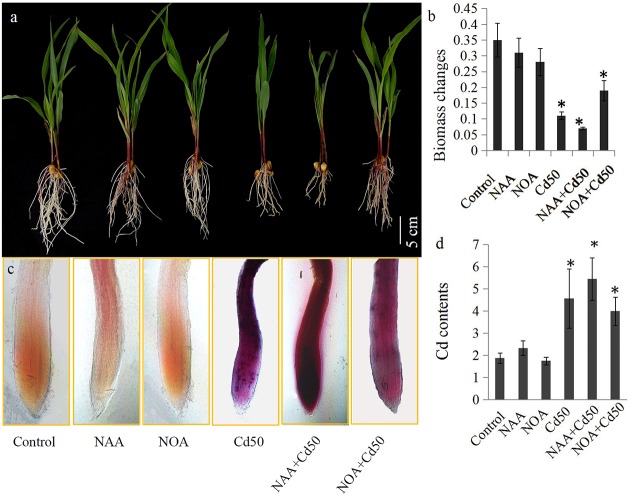
**(a) The maize seedlings were grown under different conditions, including control, NAA, NOA, Cd50, NAA + Cd50 and 1-NOA + Cd50 treatments.** The bars indicated 1 cm. **(b)** Biomass of maize seedlings under above condition with 1 μM NOA; Cd50: nutrient solution with 50 μM cadmium chloride; NAA + Cd50: nutrient solution with 0.1 ms. Control: nutrient solution without hormone and Cd; NAA: nutrient solution with 0.1 μM NAA; NOA: nutrient solution with 1 μM NOA; NAA + Cd50: nutrient solution with 0.1 μM NAA + 50 μM cadmium chloride; NOA + Cd50: 1 μM 1-NOA + 50 μM cadmium chloride. **(c)** Cd contents of maize seedlings under above conditions. The purple indicated an accumulation of Cd in roots. **(d)** Quantitative analysis of the Cd contents of maize seedlings under above condition. The data in **(c)** and **(d)** were analyzed by three independent repeats, and standard deviations were shown with error bars. Significant differences in biomass and Cd contents between control and the treated-roots were indicated by “^∗^”.

The roles of auxin signaling in root system development have been established by many previous studies (Liu et al., 2009; Wang et al., 2009; Druege et al., 2016). In our study, 0.1 μM NAA and 1 μM 1-NOA (auxin transport inhibitor) was used to examine the roles of auxin and auxin transport in the root system response to Cd stress in maize. The results indicated that an application of 50 μM Cd clearly inhibited the growth of the root system. However, root growth inhibition by 50 μM Cd was markedly alleviated by the addition of 1-NOA, but was increased by the addition of NAA (**Figure 6a**). The changes in root biomass were also measured under different conditions. These were the control, NAA, 1-NOA, Cd50, NAA + Cd50, and 1-NOA + Cd50 treatments. Low concentrations of NAA and 1-NOA did not significantly change root biomass compared to the control roots. However, 50 μM Cd caused an acute decline in maize root biomass, but this was reversed by the application of 1-NOA and increased by the addition of NAA (**Figure 6b**).

### Determination of Cd Content in Maize Roots under Different Conditions

*In situ* localization revealed the presence of Cd through the accumulation of reddish precipitates. Few reddish precipitates were observed in the three Cd-untreated roots (control, NAA, and 1-NOA treatments), but large amounts of reddish precipitates accumulated in the three Cd-treated roots (Cd50, Cd50 + NAA, and Cd50 + 1-NOA). More reddish precipitates were observed in the Cd-treated roots than in the control roots. Interestingly, more reddish precipitates were observed in the NAA + Cd50 roots than in the Cd50 roots, which suggested that Cd accumulation was high in the NAA + Cd50 roots. Furthermore, fewer reddish precipitates were observed in the 1-NOA + Cd50 roots than in the Cd50 roots, which indicated that Cd accumulation was low in the 1-NOA + Cd50 roots (**Figure 6c**). The analytical determination of Cd levels in maize roots under different conditions confirmed the *in situ* localization of Cd (**Figure 6c**).

## Discussion

Cadmium accumulation in cultivated soil has considerably increased over the last decade, and most crop plants, including maize, suffer from Cd toxicity in polluted environments (Valko et al., 2005; Anjum et al., 2015). Therefore, determining the molecular mechanisms involved in the responses to Cd stress would enable researchers to explore the potential Cd-defensive strategies that may occur in maize plants. The transcriptome data showed the differential gene expressions in ‘Zheng58’ seedlings under the control and the Cd-treated conditions. Among the DEGs between the control and Cd treatments, the overrepresented biological functional pathways genes are involved in the oxidation-reduction process, reactive oxygen species (ROS) scavenging system, and responses to stimuli, etc, which is consistent with previous studies (Ahsan et al., 2009; DalCorso et al., 2010; Lin et al., 2013).

Plants usually response to heavy metal stresses by activating the ROS system (He et al., 2015), which was the biological process that showed the greatest enhancement in activity amongst the DEGs in our data sets. In *Medicago truncatula*, Cd (100 μM) initially increased ROS and enhanced antioxidative-related enzyme activity (Rahoui et al., 2016). In moso bamboo, the activities of superoxide dismutase and peroxidase were initially enhanced after Cd addition (Li S. et al., 2016). A large number of ROS-related terms were identified and grouped into significantly differentially expressed GO terms, including ‘response to oxidative stress’ (GO:0006979), ‘oxidation-reduction process’ (GO:0055114), ‘peroxidase activity’ (GO:0004601), and ‘oxidoreductase activity’ (GO:0016705). In maize, a class III peroxidase (*ZmPRX*) gene family has been identified as a conserved plant-specific subfamily that is involved in abiotic stress responses (Wang et al., 2015). In our study, 10 *ZmPRX* genes (GRMZM2G088765, GRMZM2G047656, GRMZM2G341934, GRMZM2G050829, AC205413.4, AC211164.5, GRMZM2G085967, GRMZM2G089895, GRMZM2G043855, and GRMZM2G370928) showed significant changes between the control and Cd treatments (Supplementary Table S5), which suggested an important role for peroxidase in the Cd stress responses.

Nicotianamine (NA), which chelates iron, is a central component of plant iron homeostasis (Hell and Stephan, 2003). In *Arabidopsis*, AtNAS4 has an important role in iron distribution and is required for normal responses to Cd supply. *Atnas4*, a mutant of AtNAS4, shows enhanced sensitivity to Cd, whereas the transgenic lines overexpressing AtNAS4 were less responsive to Cd (Koen et al., 2013). Two nicotianamine synthase-related GOs, ‘nicotianamine biosynthetic process’ (GO:0030418) and ‘nicotianamine synthase activity’ (GO:0030410) were identified as significantly differentially expressed GO terms. Most of the genes that belonged to GO:0030418 and GO:0030410 were largely up-regulated by Cd treatment (Supplementary Table S6). Maize plants may partly prevent the deleterious effects of Cd by elevating endogenous NA levels.

Several previous studies have shown the effects of Cd on carbon metabolism. In bean seeds, the effects of Cd stress on carbohydrate contents (starch, soluble sugars, sucrose, glucose, and fructose) have been investigated (Sfaxi-Bousbih et al., 2010). In Rangpur lime roots, Cd accumulation increased the apoplastic sucrose content levels (Podazza et al., 2006). KEGG analysis has shown that many carbohydrate metabolism-related genes were up-regulated by Cd treatment in maize roots. Six percent of the significantly up-regulated genes were associated with the ‘starch and sucrose metabolism’ term (**Figure 3**). This suggested that carbon metabolism may play a primary role in the responses to abiotic stresses in maize seedlings. Recently, it has been shown that glutathione plays a positive role in alleviating Cd-mediated changes to different leaf sections in cotton (Daud et al., 2016). In *Sedum alfredii* Hance, the endophytic bacterium *Sphingomonas* SaMR12 promotes cadmium accumulation by increasing glutathione biosynthesis (Pan et al., 2016). In *Arabidopsis*, several genes, such as *MAN3* and *ZAT6*, regulate Cd tolerance via a glutathione-dependent pathway (Chen J. et al., 2015; Chen et al., 2016). In our study, 5% of the significantly down-regulated genes were associated with the ‘glutathione metabolism’ term (**Figure 3**). This indicated a close relationship between Cd responses and glutathione metabolism.

Previous research has shown that auxin and its transport are involved in plant responses to abiotic stress (Krishnamurthy and Rathinasabapathi, 2013). Furthermore, the auxin physiological response to Cd stress has been partially revealed in the model plant *Arabidopsis* (Hu et al., 2013). In our study, the different expression patterns of auxin-related genes, including auxin synthesis, auxin transport, and downstream auxin response genes, under Cd treatment in maize have been investigated. The expressions of most auxin-related genes were down-regulated by Cd treatment (**Figure 4**). Furthermore, IAA measurement experiment also showed that the free IAA levels were significantly reduced by Cd treatment in maize roots (**Figure 5A**), which was similar to *Arabidopsis*, poplar, pea, and *Populus* (Chaoui and El Ferjani, 2005; Elobeid et al., 2012; Lomaglio et al., 2015). In *Arabidopsis*, two important auxin synthesis-related genes, *YUC1* and *YUC5*, showed significantly reduced expressions after 48 h Cd treatment (Hu et al., 2013). In our study, four *ZmYUC* genes were identified as down-regulated Cd response genes, which is similar to the results for *Arabidopsis*. Decline in these *ZmYUC* genes may be one major cause of the reduction of IAA level in maize roots under Cd treatment. On the other hand, the alterations to endogenous IAA levels were also found to be related to IAA oxidase activity in the different groups (Chaoui and El Ferjani, 2005; Xu et al., 2009). In maize roots, IAA oxidase activity was up-regulated (**Figure 5B**), which suggested that the increase in IAA oxidase activity may contribute to the reduction in maize root auxin levels under Cd stress. Recently, Polle’s group reported that Cd treatment interferes with the metabolism of auxin in poplars by triggering increases in GH3 activities (Elobeid et al., 2012). In *Pisum sativum* L., auxin conjugate indole-3-acetyl-aspartate, which is the products of GH3 enzymes, can directly and specifically affect the responses to cadmium stress (Ostrowski et al., 2016). In our study, two GH3 genes were identified as Cd responses genes in maize, suggesting an involvement of GH3-medciated auxin homeostasis in the responses to Cd stress.

Additionally, changes in root architecture also improve the tolerance of Cd stress in plants (Yu et al., 2015; Li P. et al., 2016). In *Arabidopsis*, the primary root length was decreased under Cd stress (Hu et al., 2013). Similar inhibition of root growth was also observed in maize (**Figure 6a**). Many studies have reported that Cd stress could interfere with root growth by regulating auxin polar transporter gene expressions (Hu et al., 2013). Auxin transport influenced a common element that was involved in plant tolerance to Cd stress (Chaoui and El Ferjani, 2005; Ostrowski et al., 2016). For example, in *Arabidopsis*, Cd alleviates Cd-induced inhibition of root growth by altering the expression of auxin transport genes, such as *PIN1*, *PIN2*, *PIN4*, and *AUX1* (Hu et al., 2013; Li P. et al., 2016). The auxin transporter, OsAUX1, is involved in root system development and in Cd stress responses in rice (Yu et al., 2015). In our study, two auxin eﬄux transporter genes (*ZmPIN4* and *ZmPIN5*) and two auxin influx transporter genes (*ZmLAX1* and *ZmLAX3*) were responsive to Cd stress (**Figure 4B**). Here, we hypothesized that auxin polar transportation may play a pivotal role in Cd stress responses.

To verify this hypothesis, a synthetic auxin NAA and an auxin transport inhibitor 1-NOA were used to examine the role of auxin transportation in the root development response to Cd treatment. The results indicated that the reduction in root growth by 50 μM Cd was considerably enhanced by NAA application and was alleviated by the addition of 1-NOA (**Figure 6a**–**d**). In rice, exogenous NAA treatment improves Cd tolerance in *osaux1* mutant, the local auxin gradients provided by OsAUX1 are essential for Cd tolerance (Yu et al., 2015). The inhibition of auxin transport by 1-NOA reduced the physiological responses caused by Cd stress, suggesting a close relationship between auxin transport and the Cd stress responses.

## Conclusion

Three independent cDNA libraries from untreated roots and three independent cDNA libraries from Cd-treated roots were constructed and sequenced. A large number of DEGs were identified in maize under Cd stress. Transcription dynamics of Cd response genes and their related major biological functions were characterized based on GO and KEGG categories. Furthermore, the expression of genes related to auxin-signaling pathways was analyzed in maize, and some were validated by qRT-PCR analysis. Our data showed that auxin content and distribution were required for Cd responses in maize. Application of 1 μM 1-NOA reversed the reductions in biomass and the accumulation of Cd in maize roots. Our work may provide new molecular and physiological clues to elucidate the crosstalk between auxin transportation and Cd stress, which may help to improve production and enhance Cd tolerance of maize by regulating auxin signaling.

## Author Contributions

RY and ST designed the research. RY, CL, JQ, XH, SY, SG, and LL carried out the experiments. NC and HC analyzed the data. RY, HY, XF, and ST contributed to writing the manuscript. RY and ST supervised the project.

## Conflict of Interest Statement

The authors declare that the research was conducted in the absence of any commercial or financial relationships that could be construed as a potential conflict of interest.

## Footnotes

1 http://www.maizegdb.org/

2 http://www.dikma.com.cn
